# Transmission of *Entamoeba nuttalli* and *Trichuris trichiura* from Nonhuman Primates to Humans

**DOI:** 10.3201/eid2110.141456

**Published:** 2015-10

**Authors:** Bruno Levecke, Pierre Dorny, Francis Vercammen, Leo G. Visser, Marjan Van Esbroeck, Jozef Vercruysse, Jaco J. Verweij

**Affiliations:** Author affiliations: Ghent University, Merelbeke, Belgium (B. Levecke, P. Dorny, J. Vercruysse);; Institute of Tropical Medicine,; Antwerp, Belgium (P. Dorny, M. Van Esbroeck); Royal Zoological Society of Antwerp, Antwerp (F. Vercammen);; Leiden University Medical Center, Leiden, the Netherlands (L.G. Visser, J.J. Verweij)

**Keywords:** parasites, zoonoses, Entamoeba nuttalli, Trichuris trichiura, nonhuman primates, zoos, humans, invasive amebiasis, transmission, prevalence

**To the Editor:** *Entamoeba nuttalli* parasites are frequently found in fecal samples of nonhuman primates (NHPs) in zoos (*1*). This gastrointestinal pathogen is the causative agent of invasive amebiasis in NHPs, which may result in hemorrhagic dysentery, liver abscess or other extraintestinal pathologies, and even death (*2*). *E. histolytica* infection is the cause of invasive amebiasis in humans and can also cause experimentally invasive amebiasis in NHPs (*3*). The host specificity of *E. nuttalli* and *E. histolytica* parasites remains largely unknown. Although molecular analyses indicate that *E. nuttalli* parasites are genetically different from *E. histolytica* parasites (*4*), and hence provide a unique marker to identify zoonotic transmission, molecular tools have not been applied extensively to determine the presence of *E. nuttalli* in humans (*5*). Moreover, studies suggesting transmission have been based on clinical outbreaks in animal caretakers (*2*), and because infections may not always be symptomatic, the results of those studies may underestimate the incidence.

We conducted a study to assess the occurrence of zoonotic transmission of *E. nuttalli* and other gastrointestinal parasites in animal caretakers in 5 zoos in Belgium and the Netherlands. A previous cross-sectional survey (*6*) in these zoos indicated that >80% of the 67 groups of NHPs (40 species, 400 animals, and 1,435 samples) were infected with at least 1 of the 13 gastrointestinal parasites found. In that survey, the most frequently detected parasites were *E. nuttalli* (found in 7 [10.4%] of the 67 groups of NHPs), *Trichuris trichiura* (12 [17.9%]), *Balantidium coli* (14 [20.9%]), and *Giardia duodenalis* (26 [38.8%]). These parasites can cause clinical symptoms in animals and humans. In our study, caretakers of NHPs in each zoo were screened on a voluntary basis for gastrointestinal parasites; animals other than NHPs were also screened (control group). Fecal samples were processed by the acid-ether concentration method and then examined by microscopy to identify the presence of protozoan cysts and helminth eggs (*6*). With the exception of fecal samples from 13 caretakers at 2 zoos, all samples were also processed by using real-time PCR to ascertain the presence of *E. nuttall, E. histolytica,* and *E. dispar* parasites (*7*). *E. nuttalli* can only be detected by PCR targeting the *E. histolytica*–specific repeat; the parasite cannot be detected by a PCR based on the ribosomal small subunit of *E. histolytica*. Both PCRs amplified *E. histolytica* (*8*). Fisher exact test was applied to determine whether there was a difference in parasite infection, as determined by microscopy, between caretakers responsible for NHPs and those caring for animals other than NHPs. An independent physician gave medication to caretakers who were infected with parasites. The study protocol was approved by the Ethics Committee of Ghent University (Belgium; no. 2008/359), Antwerp University (Belgium; no. UA A08 21), and Leiden University Medical Center (the Netherlands; no. 26734).

Fifty-four animal caretakers from 5 zoos participated in the survey; 42 of the caretakers were responsible for NHPs and 12 for animals other than NHPs. Microscopy and PCR results showed that 16 (29.6%) caretakers were infected with >1 parasite. We used microscopy to detect cysts of various *Entamoeba* species in fecal samples from 13 (24.1%) caretakers; samples from 4 (7.4%) caretakers were positive for *T. trichiura* eggs. Infections with *Entamoeba* species and *T. trichiura* were observed only in caretakers of NHPs, suggesting that these caretakers are at higher risk of acquiring parasitic infections (p = 0.03). PCR detected *E. nuttalli, E. histolytica,* and *E. dispar* parasites in 1, 1, and 3 of 41 caretakers, respectively. We were not able to perform PCR on all fecal samples from 2 zoos (13 caretakers). *E. nuttalli* parasites were detected in a caretaker working in a zoo where *E. nuttalli* infections were prevalent in animals. *E. histolytica* parasites was detected in a caretaker employed in a zoo where *E. nuttalli* infections were not detected in animals. All *T. trichiura* infections were detected in caretakers from 1 zoo, and *T. trichiura* infections were also prevalent in that zoo’s animals. None of the caretakers reported gastrointestinal problems.

The presence of *E. nuttalli* and *T. trichiura* parasites in caretakers in zoos where these parasites are also prevalent in NHPs strongly suggests NHP-to-human transmission of these parasites, as was reported for *Blastocystis* parasites (*9*). Genotyping of *E. nuttalli* isolates from NHPs and animal caretakers could be performed for additional confirmation of NHP-to-human transmission of *E. nuttalli* parasites (*10*). The *E. nuttalli*–infected caretaker in this study appeared to be completely asymptomatic. Although *E. nuttalli* parasites are as virulent as *E. histolytica* parasites in animal models, it remains unclear whether they are as virulent in humans (*4*). We recommend that caretakers of NHPs be screened on a regular basis and be provided with appropriate medications if needed.
